# Two additional species of *Gymnopus* (Euagarics, Basidiomycotina)

**DOI:** 10.3897/mycokeys.45.29350

**Published:** 2019-01-14

**Authors:** Ronald H. Petersen, Karen W. Hughes

**Affiliations:** 1 Ecology & Evolutionary Biology, University of Tennessee, Knoxville, TN 37996-1100 University of Tennessee Knoxville United States of America

**Keywords:** Fungal barcode, Marasmius, Omphalotaceae, phylogenetics, taxonomy

## Abstract

For more than a decade, a combination of molecular phylogenetic analyses and morphological characterisation has led to a renovation of the Omphalotaceae, especially of *Gymnopus* sensu lato. Numerous new genera have been proposed, but *Gymnopus* sensu stricto has also seen an accretion of species and species complexes. In this manuscript, two species are added to Gymnopus sensu stricto within Section Androsacei.

## Introduction

Ongoing research on marasmioid and gymnopoid fungi (Antonín and Noordeloos 2010; Antonín et al. 2014; Mata et al. 2004; Petersen and Hughes 2016, 2017; Wilson and Desjardin 2005), has led to significant renovation of *Gymnopus* sensu lato. Several additional genera have been proposed and molecular phylogenetic analyses have revealed numerous small clades within *Gymnopus* sensu stricto. One such clade includes *Marasmiusbrevipes* Berk. & Ravenel. The result is the necessary transfer of *M.brevipes* to *Gymnopus* and proposal of a new species, *G.portoricensis*.

Nomenclaturally, recombination of *Marasmiusbrevipes* into *Gymnopus* produces a conflict between two potential homonyms, of which *Gymnopusbrevipes* (Bull.) S.F. Gray has priority. A new name is required for Marasmius (Gymnopus) brevipes and this is introduced below as *Gymnopusneobrevipes*.

## Materials and methods

The following abbreviations and acronyms are noted: RHP, KWH = initials of the authors; GSMNP, Great Smoky Mountains National Park; M = Marasmius; Ma = Marasmiellus; Mi = Micromphale; My = Mycetinis. Colour names enclosed in quotation marks (“”) are from Ridgway (1912) and those cited alphanumerically are from Kornerup and Wanscher (1967). BF = bright field microscopy; PhC = phase contrast microscopy. Microscopic structures were observed in 3% aqueous potassium hydroxide (KOH) without staining. Spore metrics are expressed as Q = the range of spore length divided by spore width; Q^m^ = mean value of Q.

All photos of microscopic structures were taken using a Q_c_ Olympus camera mounted on an Olympus BX60 research microscope fitted with phase contrast microscopy.

**Figure 1.**
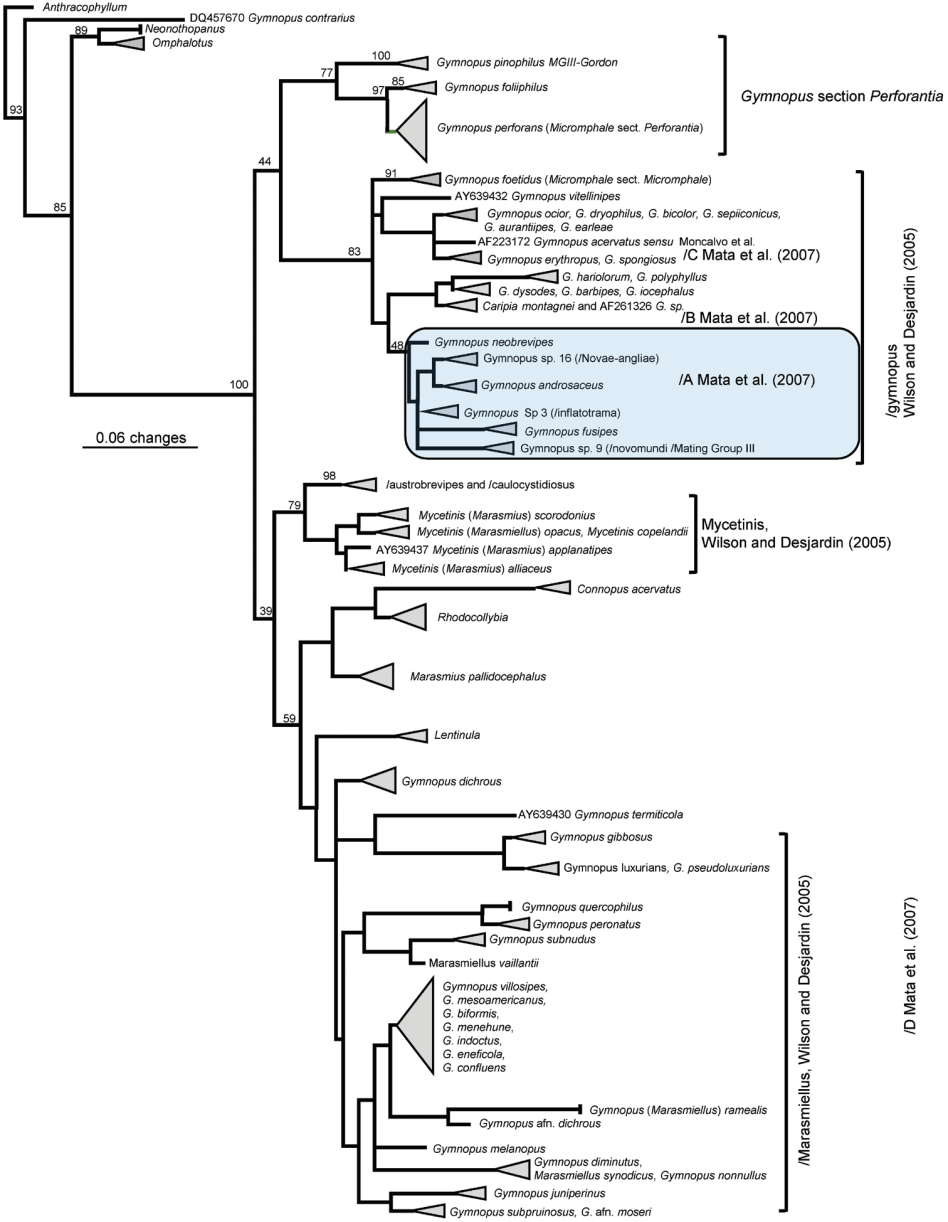
PhyML-based phylogeny of gymnopoid taxa based on nuclear LSU sequences showing the placement of *G.neobrevipes* within /gymnopus and allied with sect. Androsacei.

**Figure 2.**
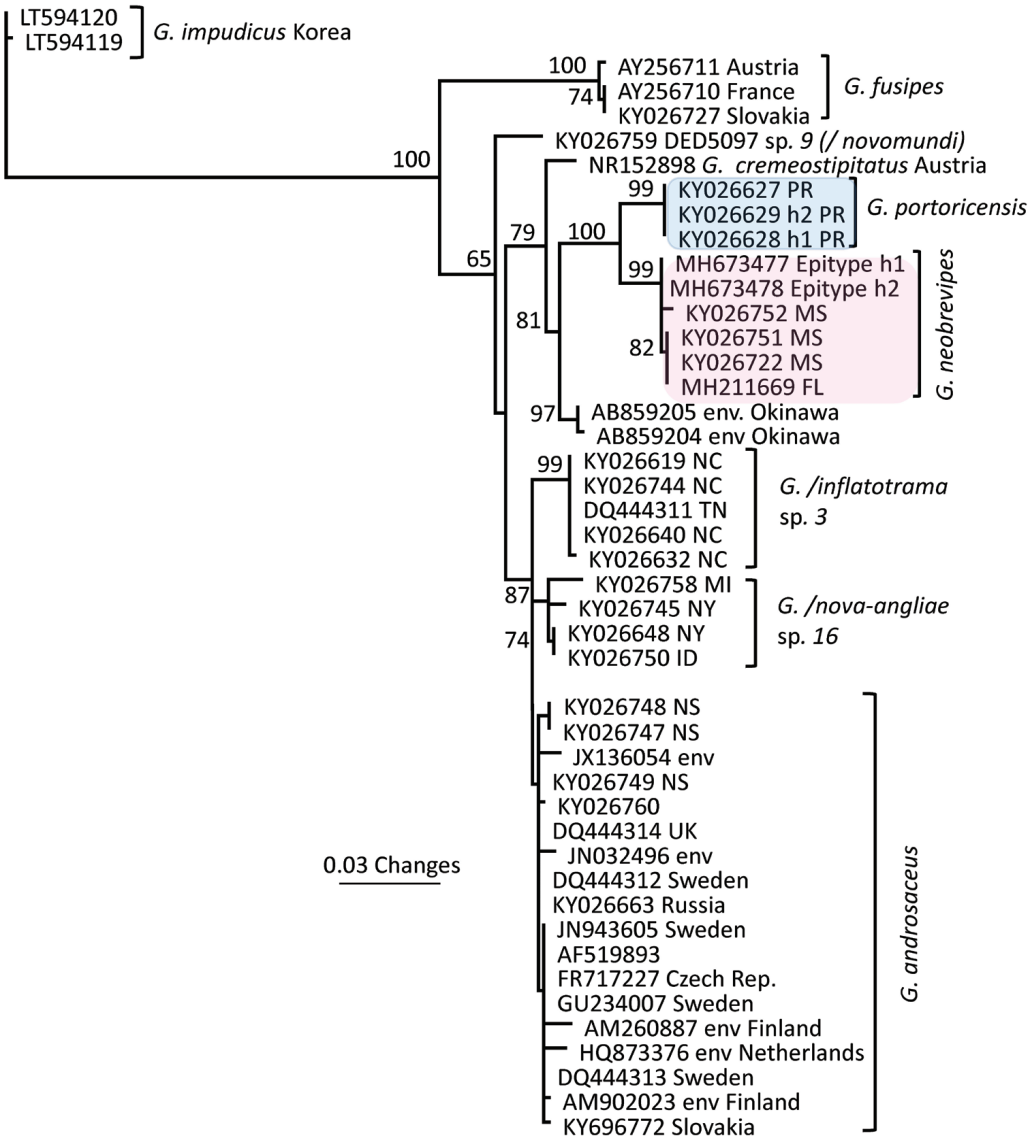
PhyML-based phylogeny based on ITS sequences, showing the relationship of *G.neobrevipes* and *G.portoricensis* to other androsaceoid taxa.

Molecular methods were described in Petersen and Hughes (Petersen and Hughes 2016; see also Petersen and Hughes 2017). An LSU-based PhyML phylogeny illustrates general placement of section Androsacei within *Gymnopus* and related taxa (Fig. 1). An ITS-based PhyML phylogeny was constructed to show more detailed placement of the two species below within Section Androsacei (Fig. 2). ITS and LSU sequences used in this paper are available in GenBank. Aligned ITS and LSU sequences are available in the Dryad depository (ITS: https://doi.org/10.5061/dryad.rd1df0c; LSU : https://doi.org/10.5061/dryad.4081h).

## Results

### Phylogenetic placement

Clade A of Mata et al. (2007) containing section Androsacei falls within /gymnopus of Wilson and Desjardin (2005) based on nuclear LSU sequences with moderate bootstrap support (Fig. 1). The small clade containing *G.portoricensis* and *G.neobrevipes* also appears in /A. At the ITS level, *G.portoricensis* and *G.neobrevipes* appear as a sister clade to *Gymnopusandrosaceus* (Fig. 2). Two environmental sequences from Okinawa and *Gymnopuscremeostipitatus* (South Korea), placed by Antonín et al. (2014) within Section Androsacei, are also related to *G.portoricensis* and *G.neobrevipes*.

### Taxonomy

#### 1. Gymnopus neobrevipes

Taxon classificationAnimaliaAgaricalesOmphalotaceae

R.H. Petersen nom. nov.

Index Fungorum no. 555346

**Figure 3.**
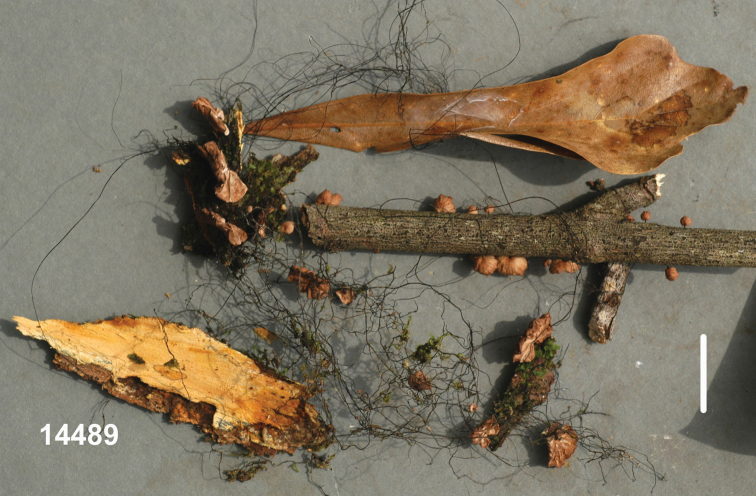
*Gymnopusneobrevipes*. Habit view. TFB 14489 (TENN-F-069182). Scale bar: 10 mm.

**Figure 4.**
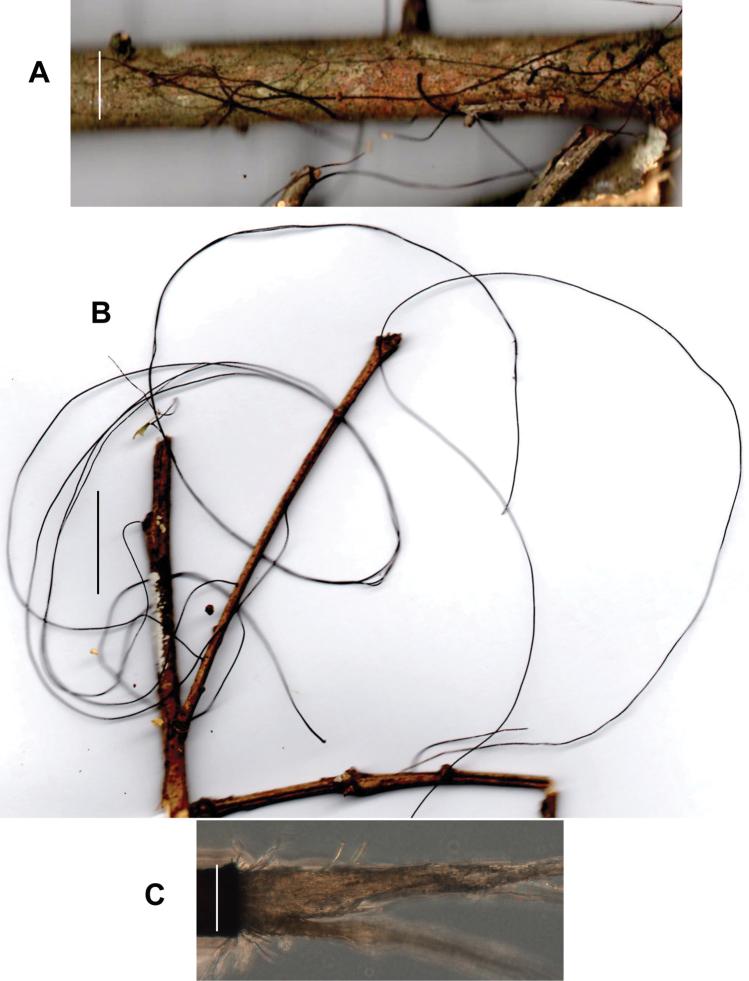
*Gymnopusneobrevipes*. **A** Resupinate rhizomorphs on surface of twig **B** Long aerial rhizomorphs on twig **C** Spray of hyphae from cut end of rhizomorph; 24 h. Rhizomorph on left. Scale bars: 10 mm (**A, B**); 1 mm (**C**). TFB 14607 (TENN-F-063931).

**Figure 5.**
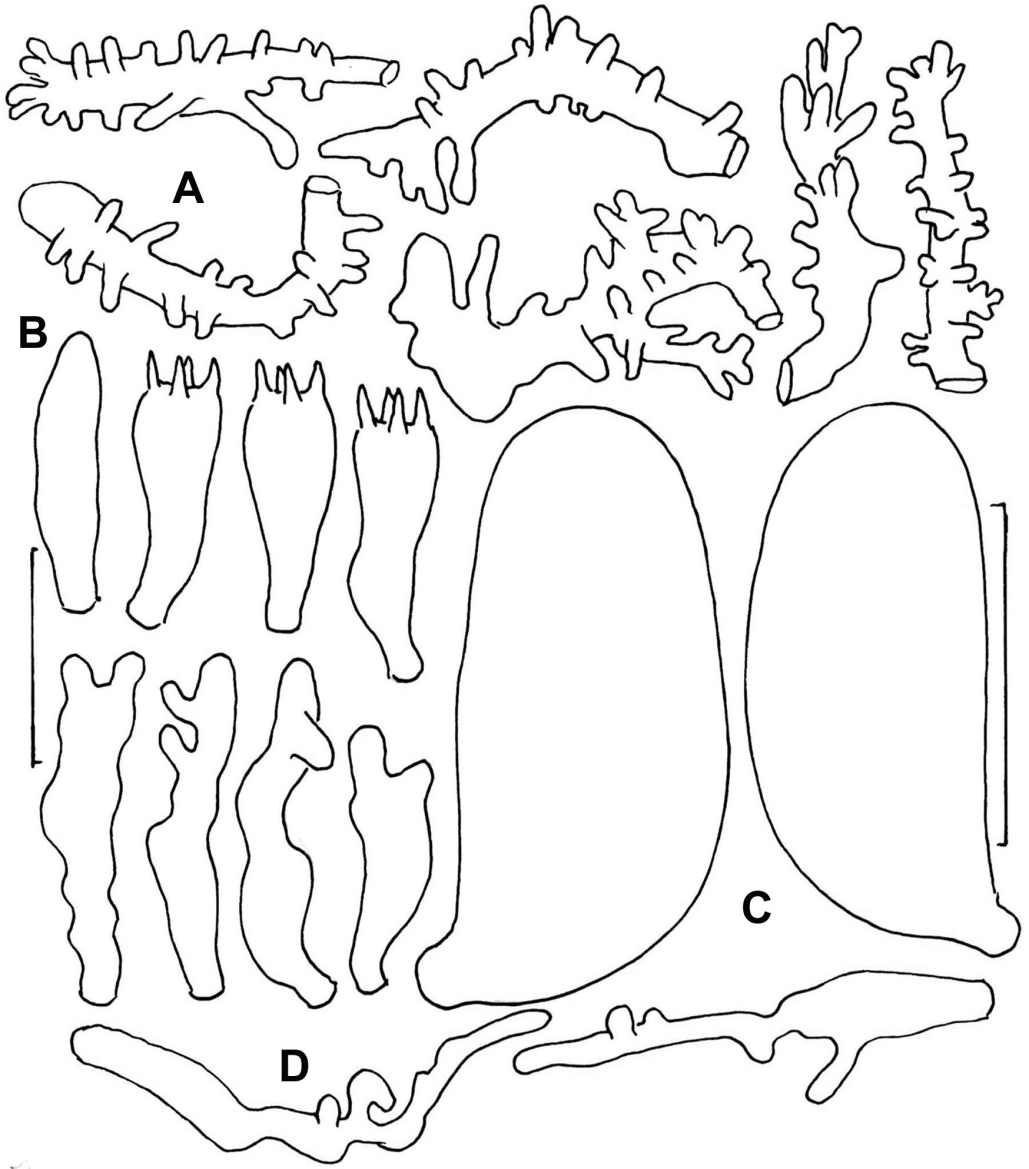
*Gymnopusneobrevipes*. Micromorphological structures. **A** Diverticulate hyphae of pileipellis **B** Pleurocystidium and basidia **C** Basidiospores **D** Cheilocystidia. **A–C** TFB 9087 (TENN-F-054912) **D** DED 4367 (TENN-F-047662). Scale bar: 20 µm (**A, B, D**); 5 µm (**C**).

**Figure 6.**
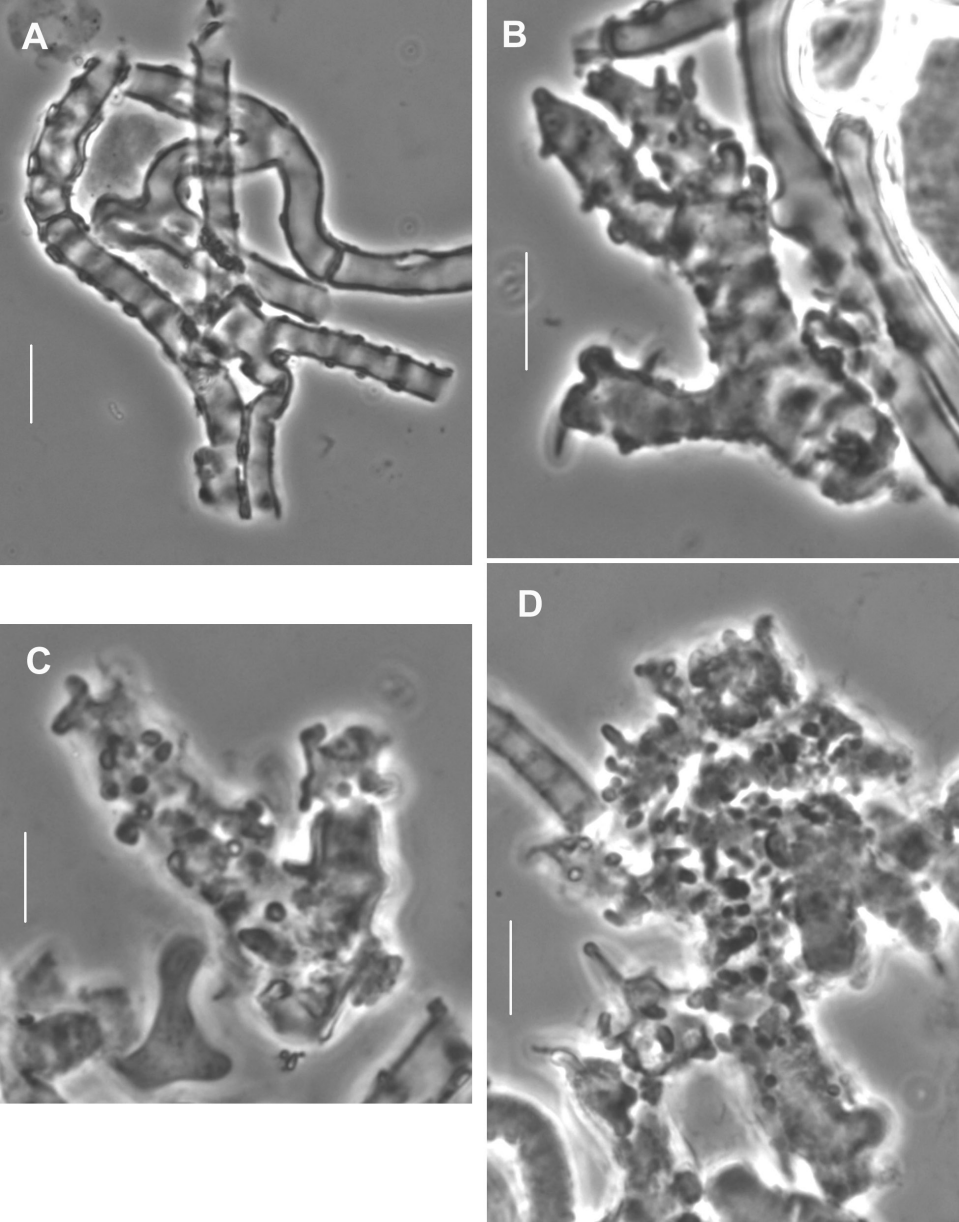
*Gymnopusneobrevipes*. Pileipellis elements. **A** Encrusted hyphae with banded appearance **B–D** Diverticulate hyphae of ramealis-structure. Scale bars: 5 µm. TFB 14607 (TENN-F-069310).

**Figure 7.**
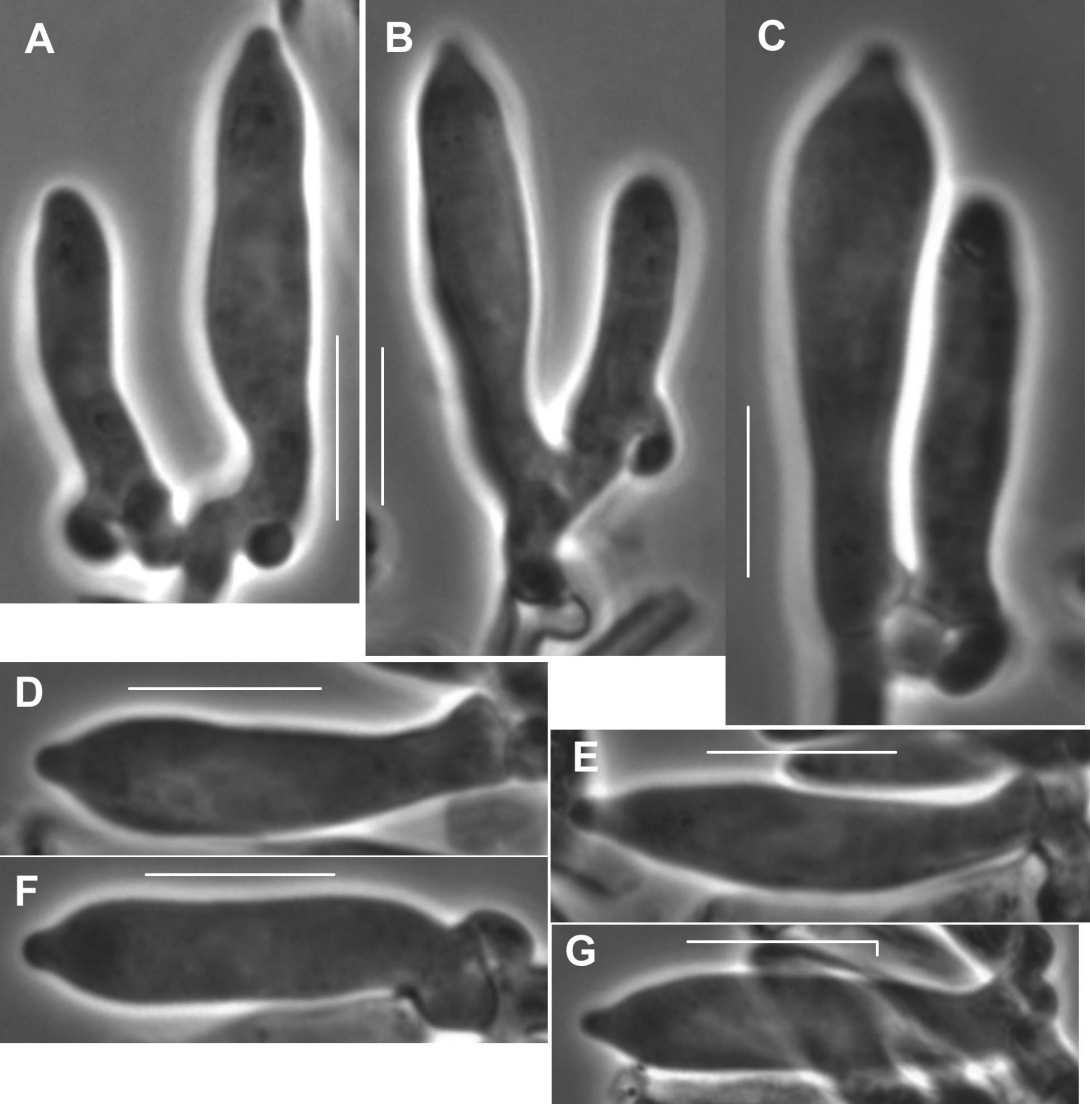
*Gymnopusneobrevipes*. Pleurocystidia. **A-C, F, G** showing subtending clamp connections. Note fusiform-submammilate shape. TFB 3704 (TENN-F-050692). Scale bars: 10 μm.

**Figure 8.**
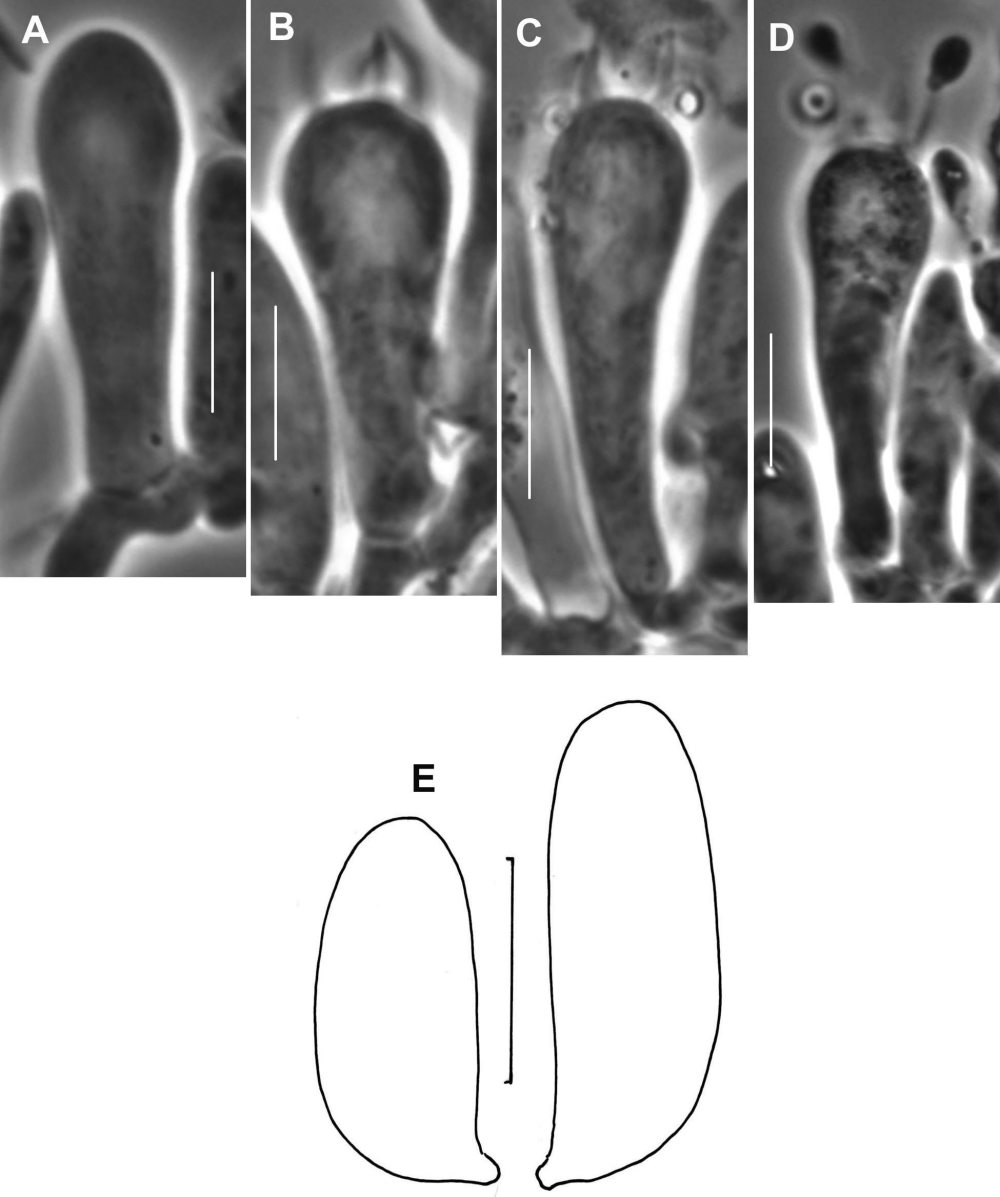
*Gymnopusneobrevipes*. A. Basidiole. **B–D** Basidia. E. Basidiospores. TFB 3704 (TENN-F-050692). Scale bars: 10 μm (**A–D**); 5 μm (**E**).

**Figure 9.**
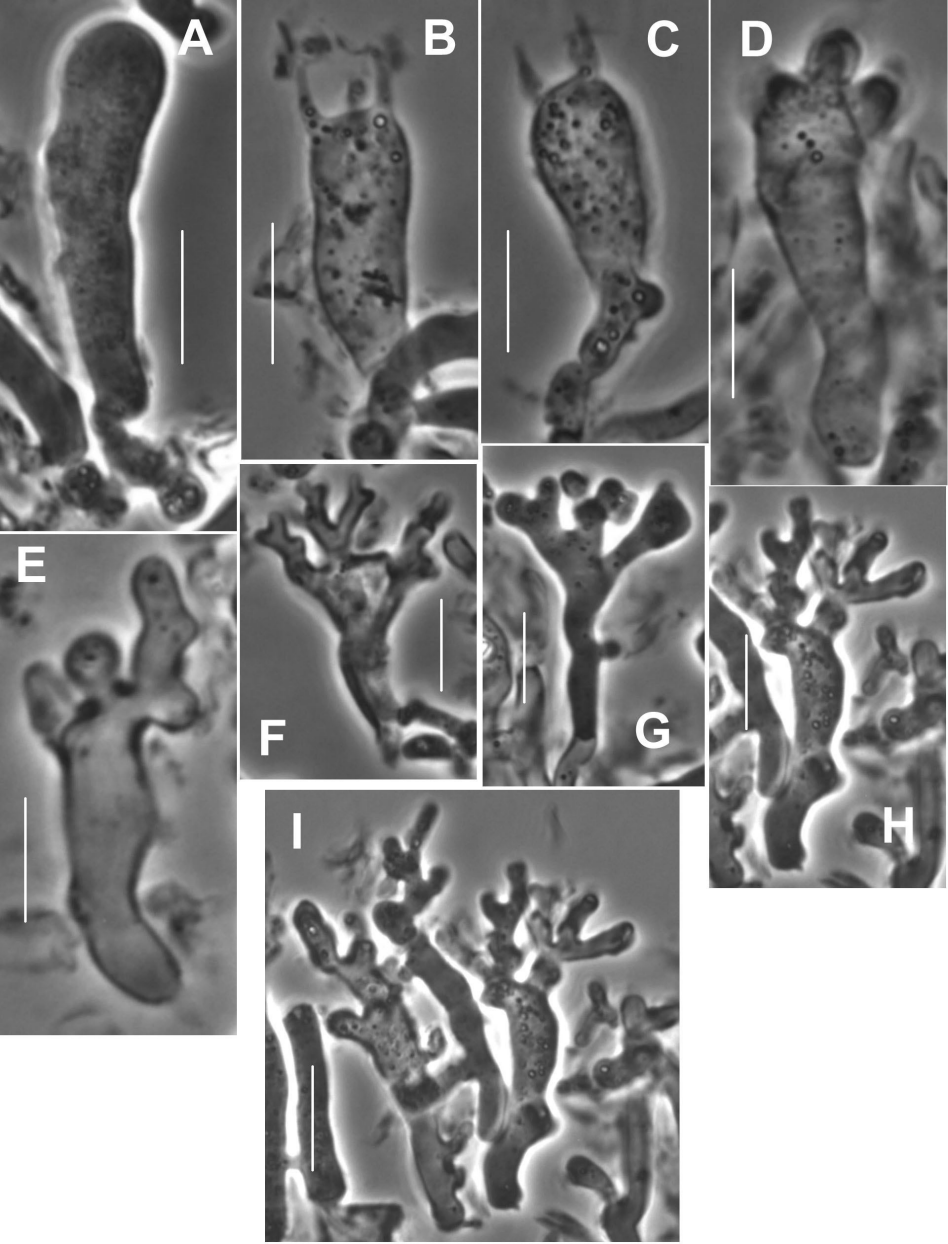
*Gymnopusneobrevipes*. **A** Basidiole **B, C** Basidia **D, E** Basidiiform cheilocystidia **F–H** Diverticulate cheilocystidia **I** Cluster of diverticulate cheilocystidia. DED 4367 (TENN-F-047662). Scale bars: 10 μm.

Figs 3 , 4 , 5 , 6 , 7 , 8 , 9

≡ Marasmiusbrevipes Berk. & Ravenel in Berkeley & Curtis. 1853. Ann. Mag. nat. Hist., Ser. 2 12: 426.
≡ Micromphalebrevipes (Berk. & Ravenel) Singer in Dennis. 1953. Kew Bull. 8(1): 42
[NOT Agaricusbrevipes Bulliard. 1791. Herbier Fr. (Paris) 11: tab. 521 (with legend); ≡ Gymnopusbrevipes (Bull.) Gray. 1821. Nat. Arr. Brit. Pl. (London) 1: 609, pre-occupied homonym] (See *Index Fungorum* for additional combinations of Bulliard’s epithet)
≠ Gymnopuswestii (Murrill) César et al. 2018 Mycokeys 42: 31. (Basionym: Marasmiuswestii Murrill. Proc. Florida Acad. Sci. 7:110. 1945.

##### Holotype.

United States, South Carolina, Santee Canal, June, Ravenel no. 1527, on dead twigs of oak (K). Type studies: Dennis 1953; Desjardin 1989; Desjardin and Petersen 1989.

##### Epitype

**(IF no. 555711)** Mississippi, George Co., Pascagoula Wildlife Management Area, vic. Boat Ramp off Rte. 26, 30°53.789'N, 88°44.848'W, 12.VII.2014, coll. KWH, TFB 14505 (TENN-F-069197). GenBank: MH673477-8.

##### Diagnosis.

1) Long, hair-like rhizomorphs usually common to dominant; 2) basidiomata small (pileus usually <10 mm broad), arising from woody substrates or as branches of rhizomorphs; 3) clamp connections ubiquitous; 4) stipe short (<5 mm long), often strongly curved; 5) stipe medullary hyphae coherent; 6) pileipellis elements usually semi-gelatinised; 7) south-eastern United States.

##### Description.

**Basidiomata** (Fig. 3) small with very short stipe, sometimes appearing resupinate or pseudostipitate (but not so), arising directly from substrate twig usually in fissures in thin bark or as side branches of extensive, black, interwoven rhizomorphs which often occur without associated basidiomata. **Pileus** 2–6(–9) mm broad, at first convex to conchate, usually becoming plano-convex or applanate by maturity, often folding closed like a clam-shell upon drying, matt, often strongly sulcate-striate almost to centre, irregularly corrugate or tuberculate, very thin but pliable; disc “burnt umber” (7E7) to “wood brown” (7C4); limb near “pinkish-cinnamon”(7B5), “avellaneous” (7B3), “wood brown” (7C4), “fawn colour,” sometimes brown (7E5-7) to dark brown (7F5) to light brown (6D-E5-6) or brownish-orange (6C4) overall or with pale striations; margin even when young, wavy in age, not striate, sometimes pale to “tilleul buff” (7B2) ; pileus flesh thin, tough, pliable. **Lamellae** adnate, distant to very distant, shallow, fold-like to sublamelloid, thickish, occasionally weakly anastomosing, “tilleul buff” (7B2), “pale pinkish cinnamon” (6A2), pale brown (7D4), “avellaneous” (7B3), “vinaceous cinnamon” (7B4), usually becoming brownish, “sayal brown” (6C5) upon drying and storage; short lamellulae common. **Stipe** 0.5–6 × 0.5–1.5 mm, more or less terete, usually equal, central, strongly ageotropic (more or less straight when occurring on upper surface of substrate, strongly curved when occurring on vertical surface, almost pseudostipitate when occurring on lower surface of substrate), glabrous to unpolished, “fawn colour” (7C5), “army brown” (8D5) “fuscous” (6E4), “burnt umber” (7E7) to dark reddish-brown (8F6-8), black at base; insertion broad with minute, brown basal tuft, usually associated with small fissures in thin bark, rarely as a side branch of aerial rhizomorph; adventitious “stipes” occasionally hypertrophic and then clavate to fusiform. **Rhizomorphs** (Figs 3, 4A, B) rarely absent, usually dominant, –80(–450) × 0.3–0.6 mm, hair-like, matt to glabrous but not polished, black, tough, occasionally branched with spur branches, rarely anastomosed, but commonly braiding so as to appear thicker than individually, ranging from resupinate on woody substrate (black, adhering to substrate by minute fringe of brown hyphae) to producing ascending individuals (and then somewhat more slender than resupinate individuals), often colonising suspended leaves and twigs to form a substrate net, occasionally producing basidiomata on side branches, often 3–4 in a file. **Taste** negligible or weakly alliaceous (reportedly weakly krauty); **odour** negligible.

##### Habitat and phenology.

Basidiomata on dead small branches of broad-leafed trees, in temperate forests often on fallen branches of *Quercus* or *Rhododen*dron m*aximum* in mixed forest including *Tsuga*, usually at or near ground level; sterile rhizomorphs decumbent on dead, small (usually 18–24 mm diam.) boughs. In tropical climates, (see Pegler 1983, 1987, 1988, Dennis 1953, 1970) encountered year-round; in temperate forests mid-Summer to early Autumn. *Gymnopusneobrevipes* sometimes shares the same habitat as *Anthracophyllumlateritium* (Berk. & Curtis) Singer – dead *Rhododendronmaximum* boughs over streams.

##### Pileipellis

composed of four elements: 1) slender “pileal hairs” occasional, 2.5–4.5 µm diam., hyaline, minutely decorated with “flakes,” usually subcapitate; capitulum often decorated with minute needle-like crystals; 2) diverticulate hyphae (Figs 5A, 6B–D) inflated -10 µm diam., thin-walled, beset with vermiform setulae, with parent hypha often subgelatinised but with setulae remaining; 3) non-orientated, repent hyphae (Fig. 6A) 3–6 µm diam., firm-walled, involved in some slime matrix, encrusted to varying degrees (from conspicuous stripes or rings with plate-like profile calluses, to flat profile calluses with only “shadow” stripes or none at all); these hyphae (overnight in KOH) tend to gelatinise walls, apparently without clamp connections; and 4) more or less erect, modified broom structures, extremely rare and usually partially gelatinised, composed of a stalk (usually with flake-like scabs) and complex series of branchlets ending in the digitate diverticulate processes, often dichotomous, as in a Rameales-structure. Pileus trama (and lamellar trama) loosely interwoven; hyphae 3–18 µm diam., firm- to thick-walled (wall -1.0 µm thick, hyaline), occasionally but conspicuously clamped, encrusted with minute debris and embedded in thin slime matrix. **Pleurocystidia** (Figs 5B, 7) common, 18–24 × 5.6–7.5 μm, fusiform to fusiform-mammilate, conspicuously clamped. Basidioles (Figs 5B, 8A) clavate, occasionally subcapitulate; **basidia** (Figs 5B, 8B–D, 9B–C) 24–27 × 7–9 µm, clavate, 4-sterigmate, obscurely clamped; contents minutely multiguttulate when mature. **Basidiospores** (Figs 5C, 8E) (6.5–)8–9 × 3.5–4(–4.5) µm (Q = 1.86–2.29; Q^m^ = 2.08; L^m^ = 8.10 µm), ellipsoid to plump-ellipsoid, flattened adaxially, smooth, thin-walled, inamyloid; contents vaguely univacuolate. **Cheilocystidial structures** (Figs 5D, 9D–I) locally common to absent but apparently only close to lamellar edge, stalked or from basidiolar cells which grow out, often appearing as though arising deeper in lamellar trama than hymenium, lobed or subdiverticulate, 2.5–3.5 µm diam., thin-walled, hyaline, obscurely clamped. **Stipe** medullary hyphae 2–7 µm diam., hyaline, thick-walled (wall -1.5 µm thick), strictly parallel, coherent, with scattered conspicuous clamp connections. Stipe cortical hyphae 3.5–7.5 µm diam., pigmented olive-brown (PhC), subdextrinoid, thick-walled (wall -2.5 µm thick), smooth, rarely perhaps producing an ampulliform side branch with extended apex (two seen). **Rhizomorph** medullary hyphae 2–5.5(13) µm diam., thin- to firm-walled, strictly parallel and perhaps embedded in slime to be coherent, with occasional lateral branches lobate to digitate, hyaline, conspicuously clamped. Rhizomorph cortical hyphae (surface) 2–5.5(–7.5) µm diam., thick-walled (wall -0.7 µm thick to obscuring cell lumen, weakly pigmented olive tan – deep olive-brown in mass; PhC), outer wall roughened with scabs of encrustation; profile calluses -0.6 µm thick, somewhat darker than hyphal walls.

##### Commentary.

Although collected by Ravenel, it was Curtis who conveyed the type specimen to Berkeley and Berkeley is the name-giver. The protologue (assumedly written by Berkeley) is in Berkeley and Curtis 1853: 426. “29. *Marasmiusbrevipes*, Berk. & Rav. MSS. Pileo convexo estriato atro-sanguineo; stipitate brevi filiformi aterriimo nitida e mycelio repente similari enato; lamellis paucis adnatis rufis. Rav. No. 1527. On dead twigs of oak, June, Santee Canal, South Carolina, H.W. Ravenel, Esq.

“Pileus 1–2 line broad, convex, dark blood red; margin even; stem filiform, jet black, quite smooth, 1–2 line high, springing from creeping mycelial thread of the same nature with itself; gills ventricose, few, adnate, rufous.

“Allied to *M.haematocephalus*, &s, but distinguished at once by its short polished stem and dark gills. The colour of the pileus is nearly that of *M.atrorubens*.“

The pileipellis structure is similar to others described in sect. Androsacei. Desjardin and Petersen (1989) described pileipellis as not gelatinised (the tissue is not so), but failed to describe the gelatinisation of individual hyphae. This gelatinisation is merely a minor gelatinous sheath of individual hyphae for the outline of hyphal wall is not altered. However, the flake-like encrustation is carried on the gel surface and is seen at some small distance from the hyphal wall outline.

Amongst basidia in a mount soaked in KOH overnight, structures are seen which can be interpreted as gelatinised cheilocystidia. In rare cases, the remnants of digitate branching can be seen, but usually nothing is left of the supporting cell but some ghost-like structure. In a mount of lamellar edge only briefly in KOH, an enormous amount of debris is detected surrounding hymenial structures. It appears to be some sort of degeneration, quite possibly partial gelatinisation, but including numerous rod-shaped bacteria. This may be another indication of gelatinisation of tissues, this time of old basidia and subhymenial hyphae.

Subbasidial hyphae (subhymenium) become zig-zag in form as basidia are formed, evacuate and disappear. These hyphae are easily mistaken for some sort of cystidial structures, especially cheilocystidia.

A chronology of authoritative literature follows. Singer (in Dennis 1953) supplied a detailed description of *G.neobrevipes* (as *Mi.brevipes*) and Dennis (1953) examined type material and offered a rather uninformative illustration. Dennis (1970) offered a description of *M.brevipes* (as *Micromphale*), but perhaps as valuable is a diminutive aquarelle (Pl. 8, Fig. 4) which provides a good representation. Perhaps the best description of *G.neobrevipes* (as *Mi.brevipes*) was offered by Pegler (1983) and the description was based on more specimens than the type. Desjardin (1989: 447–449) examined the type of *M.brevipes* and Desjardin and Petersen (1989) published a species description based on numerous specimens.

Pileipellis structures, especially erect broom cell-like cells, are often gelatinised, especially in age. Likewise, cheilocystidia, while observed only occasionally, are often reduced to debris by gelatinisation or occasionally produce apical growths which can attain significant length. Lamellar tramal hyphae are often observed as thick-walled, but usually this is due to gelatinisation of the hyphal walls (inner wall boundary is clear, but outer wall boundary is obliterated and the gelatinised wall appears as though thick).

Pegler (1983) included *Mi.brevipes* as the only representative of *Micromphale* in the Lesser Antilles.

Rhizomorphs of *G.neobrevipes* are viable and short surface-sterilised sections (circum 1 cm) placed on malt extract agar produce sprays of mycelium from severed ends within 24 hrs. Within 72 h, the emergent mycelial sprays can be excised to establish an independent dikaryon culture which can be used for sequencing. In the case of *G.neobrevipes*, not only are sprays of mycelium produced on the cut ends (Fig. 4C), but within 72 h, many lateral hyphae emerge from the rhizomorph surface, soon resembling brownish fur. In one case, rhizomorphs of an ambient air-dried specimen were stored for over a year, yet produced mycelium as noted. In another case, a collection was heat-dried, but over one month later, rhizomorphs remained viable.

In Desjardin and Petersen (1989), a comparison was made of *M.brevipes* Berkeley & Ravenel, (1853; type, South Carolina, K) to *Marasmiusporphyreticus*Petch (1947; type, Sri Lanka, K). They concluded that *M.porphyreticus* “differs mainly in absence of cheilocystidia and in forming ‘plicatosulcate’ pilei.” Other discriminating characters of *M.porphyreticus*: “slightly thinner pileus context, more regularly forked lamellae, and basidiomata not arising directly from rhizomorphs.” Significantly, presence or absence of clamp connections was not mentioned. Plicatosulcate pilei, forked lamellae and basidiomata arising from rhizomorphs are all found in *G.neobrevipes* as well as other similar basidiomata. The comparison, despite a disparate geographic distribution, remains questionable, possibly pending phylogenetic data.

Likewise, it may be necessary to compare *G.neobrevipes* to *Marasmiustomentellus* Berk. & M.A. Curtis. “1868” (1869). J. Linnaean Soc. Bot. 10(no. 45): 298 [≡ *Gymnopustomentellus* (Berk. & M.A. Curtis) Tkalčec & Mešec. 2013 Mycotaxon 123: 428], a taxon not mentioned by Desjardin and Petersen (1989).

Berkeley and Curtis’s protologue: “Pileo convexo sulcato fulvo, stipite communi nigro albo-pubescente; stipitibus fertilibus brevibus pubescentibus; lamellis paucis pileo concoloribus. On dead wood. Pileus 1 line (~2 mm) across; fertile stems 2 lines (4–5 mm) high. Common on stems many inches long. Wright 22, Herb. Berk. This is a rhizomorphic species of *Marasmius*.“ (Cuba, holotype K).

Pegler (1987), with access to the type specimen of *M.tomentellus*, wrote: “This is a rhizomorphic species of Marasmius belonging to the section Androsacei Kuehner. The minute basidiomes, consisting of a pileus, 1–3 mm diam., with a short stipe, 1–4 × 0–2–0–4 mm, are borne on a common, slender rhizomorph, also about 0.2–0.4 mm diam. The stipe and rhizomorph surfaces are characterized by a white pubescence formed by numerous, short, thick-walled, hyaline hairs, 35–120 × 3–7 μm. The pileipellis is formed of irregular, diverticulate, hyaline elements, 10–17 × 4–12 μm. Only one collapsed spore, measuring 10 × 3–5 μm, could be found on the slide preparation taken from the type specimen. Singer (1976: 79) found spores on material from Louisiana, USA, which measured 11–11–5 × 4.5 μm, oblong to cylindric, but this material was not part of the type. This tiny species was well illustrated by Dennis (1951)” Presence or absence of clamp connections was not mentioned.

Pegler (1988) did not take up *M.brevipes* as part of the Cuban mycota, but did place *M.tomentellus* in the key. From this and other literature in which the type specimen of *M.tomentellus* was examined, the following characters can be gleaned: “Pileipellis with well-developed *Rameales* structure, not truly hymeniodermic; basidiome arising from a black rhizomorph; pileus 1–3 mm diam, fulvous; rhizomorph pubescent with thick-walled, hyaline hairs; spores 10 × 3.5 µm, elongate lacrymoid (see B&C 298; I: 573 (Sect. Androsacei).”

From Desjardin’s (1989) examination of type material of *M.tomentellus*, the following can be extracted: coarse rhizomorphs with white pubescence; common short branches resembling disarticulated stipes. Only one pileus remains. Stipe and rhizomorph cortical tissue of repent hyphae -6.5 µm, thick-walled (wall -1.5 µm thick), parallel, cylindric, incrusted with granulose or amorphous brown (pigment intraparietal as well as incrusting), dextrinoid; medullary hyphae 4–8 µm diam, subparallel, cylindric, smooth, hyaline, weakly dextrinoid, thin-walled, unclamped. Rhizomorph vesture of numerous, erect rhizocystidia, 45–120 × (6–)8–12 µm, cylindric or lanceolate, obtuse or subacute, aseptate or with one or several secondary septa, apex of cell hyaline, base of cell hyaline, pale ochraceous or pale brown, weakly dextrinoid.” This appears to be the only mention of clamp connections – absent – but confirms the tomentose surface of stipes and rhizomorphs. A thorough search for tomentosity on rhizomorphs and/or stipes of collections of G. *neobrevipes* failed to observe this.

Additional species have been described in Marasmiussect.Androsacei from South-Sea Islands and at least *M.aurantiobasalis* Desjardin & Horak, (see Desjardin et al. 2000; not Desjardin and Horak 1997) exhibits several characters resembling those of *G.neobrevipes*. Finally, Tkalčec and Mešić (2013) transferred two Corner species of *Marasmius* to Gymnopussect.Androsacei. Several additional species epithets were transferred, including *M* (*Gymnopus*) *tomentellus.*

##### Specimens examined for this study.

Note that the list is not related to that offered by Desjardin and Petersen (1989): **Alabama**, Baldwin Co., Blakely Historical Park, Nature Sanctuary, 30°44'36.46"N, 87°54'56.46"W, 10.VI.2005, coll J.L. Mata, JLM 1628 (USA); same data, JLM 1630 (USA); Schillingers Rd., Cottage Hill Park, 18.VI.2004, coll D.H. Nelson, det JL Mata, JLM 1564 (USA); Mobile, Univ. South Alabama North campus, forest park, 30°41'35.06"N, 88°10'55.54"W, 3.VI.2005, coll & det J.L. Mata. JLM 1616 (USA). **Louisiana**, St. Tammany Par., vic. Pearl River, Honey Island Swamp, 6.VI.1976, coll. W.B. & V.G. Cooke, Cooke no. 52125, ex DAOM 193773 [TENN-F-054662[. [no TFB number]; See also references to Singer (in Dennis 1953). **Mississippi**, George Co., Pascagoula Wildlife Management Area, vic. Boat Ramp off Rte. 26, 30°53.789'N, 88°44.848'W, 12.VII.2014, coll. RHP, TFB 14504 (TENN 69196); same data, coll. KWH, TFB 14505 (TENN 69197); Harrison Co., vic. Saucer, Tuxahatchie Hiking Trail trailhead, 30°39'43.61"N, 89°08'14.70"W, 10.VII.2014, coll. RHP, TFB 14489 (TENN-F-069182); Red Creek Wildl. Man. Area, 11.VIII.2014, coll. KWH, TFB 14498 (TENN-F-069189); Jackson Co., Parker Lake area, Pascagoula River, 17.VII.1987, coll DE Desjardin, DED 4367 (TENN-F-047662). **North Carolina**, Macon Co., vic. Highlands, Bull Pen Rd., Slick Rock campground, 27.VII.1978, coll RHP, TFB 52193 (TENN-F-041215); vic. Highlands, Bull Pen Rd., Chattooga Loop Trail, 13.VI.1987, coll RHP & E Horak, det. DE Desjardin, DED 4279 (TENN-F-047665); same location, 13.VII.1988, DED 4583 (TUNN-F-054661); vic. Highlands, Horse Cove Rd. opposite FR 401, 13.VI.1989, coll RHP, TFB 56693 (TENN-F-048667); vic. Highlands, Nantahala Nat. For., Blue Valley, first gated road on left, 24.VI.1989, coll. RHP, TFB 1827 (TENN-F-048533); same location, FS79, 8.VII.1990, coll. RHP, TFB 2895 (TENN-F-049257); same location, 10.VII.1990, coll RHP, TFB 2185 (TENN-F-048796); same location, 10.VII.1990, coll RHP, TFB 2187 (TENN-F-048794); same location, Pickelseimer’s Falls trail, 18.VII.1991, coll. S.A. Gordon, TFB 3704 (TENN-F-050692; same location, junction of F.R. 83 and 83B, 14.VII.1986, coll D.E. Desjardin, DED 3813 (TENN-F-047663); same location, 13.VI.1987, coll RHP & E. Horak, det. DE Desjardin, 13.VI.1987, DED 4282 (TENN-F-047664). **Tennessee**, Cocke Co., GSMNP, Big Creek, 35°46'51.96"N, 83°12'11.74"W, 16.VI.1991, coll SA Gordon, RHP, V Antonin, HR Bhandary, TFB 3633 (TENN-F-050752); same location, 16.VI.1991, same collectors, TFB 3634 (TENN-F-050753). **Texas**, Hardin Co., Big Thicket Nat. Preserve, Lance Rosier Unit, Teel Rd., vic cypress swamp, 30°15.860'N, 94°30.75'W, coll DP Lewis, DPL 11773, TFB 14609 (TENN-F-069312); Newton Co., Co. Rd. 305, Bleakwood, Lewis Properties, 30°42.509'N, 93°49.630'W, coll & leg D.P. Lewis, DPL 11763 (DPL Herb.)

#### 2. Gymnopus portoricensis

Taxon classificationAnimaliaAgaricalesOmphalotaceae

R.H. Petersen sp. nov.

Index Fungorum no. IF555347

**Figure 10.**
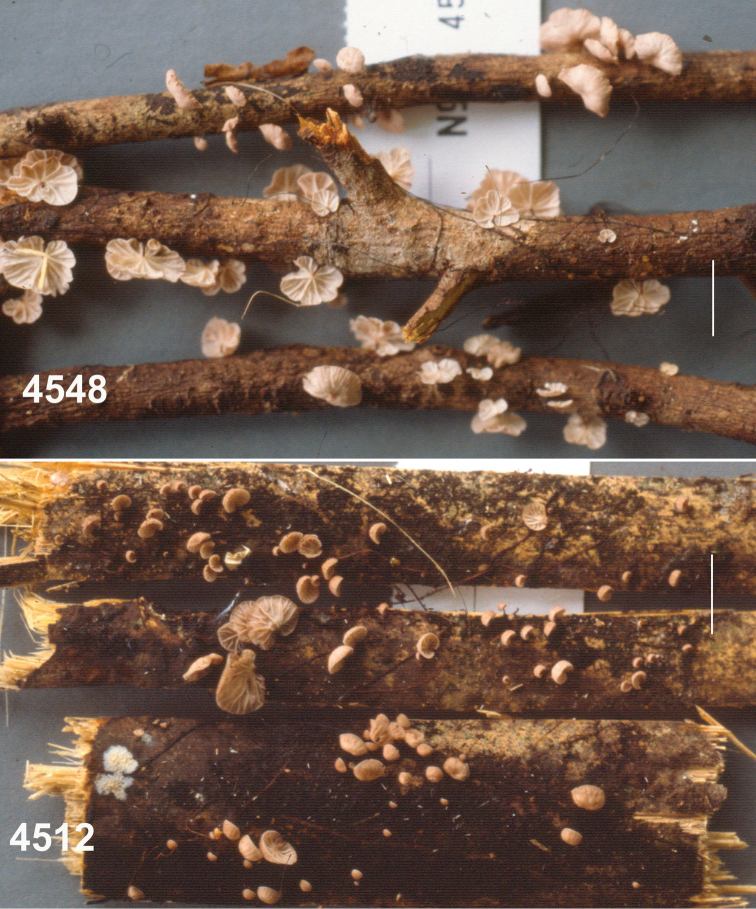
*Gymnopusportoricensis*. Habit. Above: TFB 4548 (TENN-F-051029). Below: TFB 4512 (TENN-F-050999). Scale bars: 10 mm.

**Figure 11.**
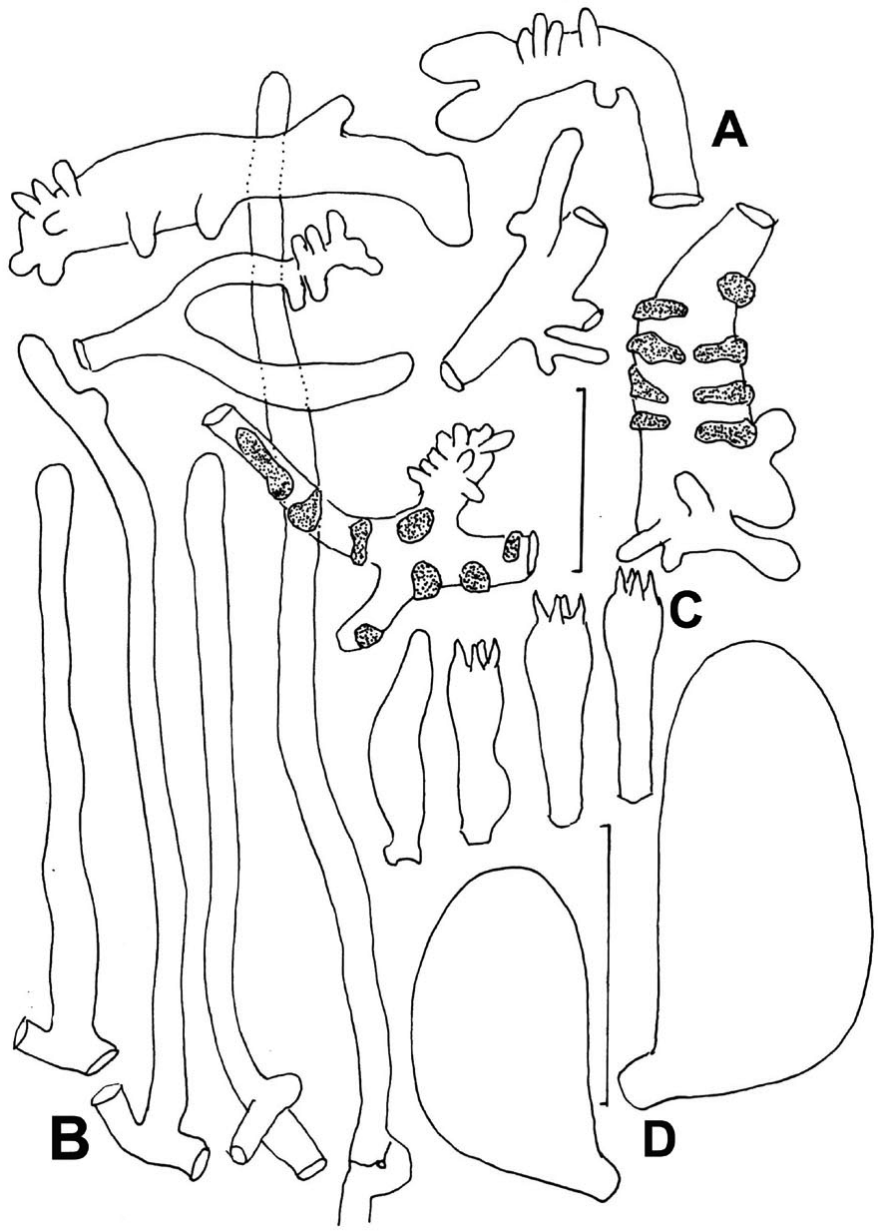
*Gymnopusportoricensis*. Microstructures. **A** Pileipellis structures; diverticulate and encrusted hyphal termini **B** “Pileal hairs.” **C** Pleurocystidium and basidia **D** Basidiospores. Scale bars: 20 µm (**A–D**); 5 µm (**E**). TFB 4548 (TENN-F-051029).

**Figure 12.**
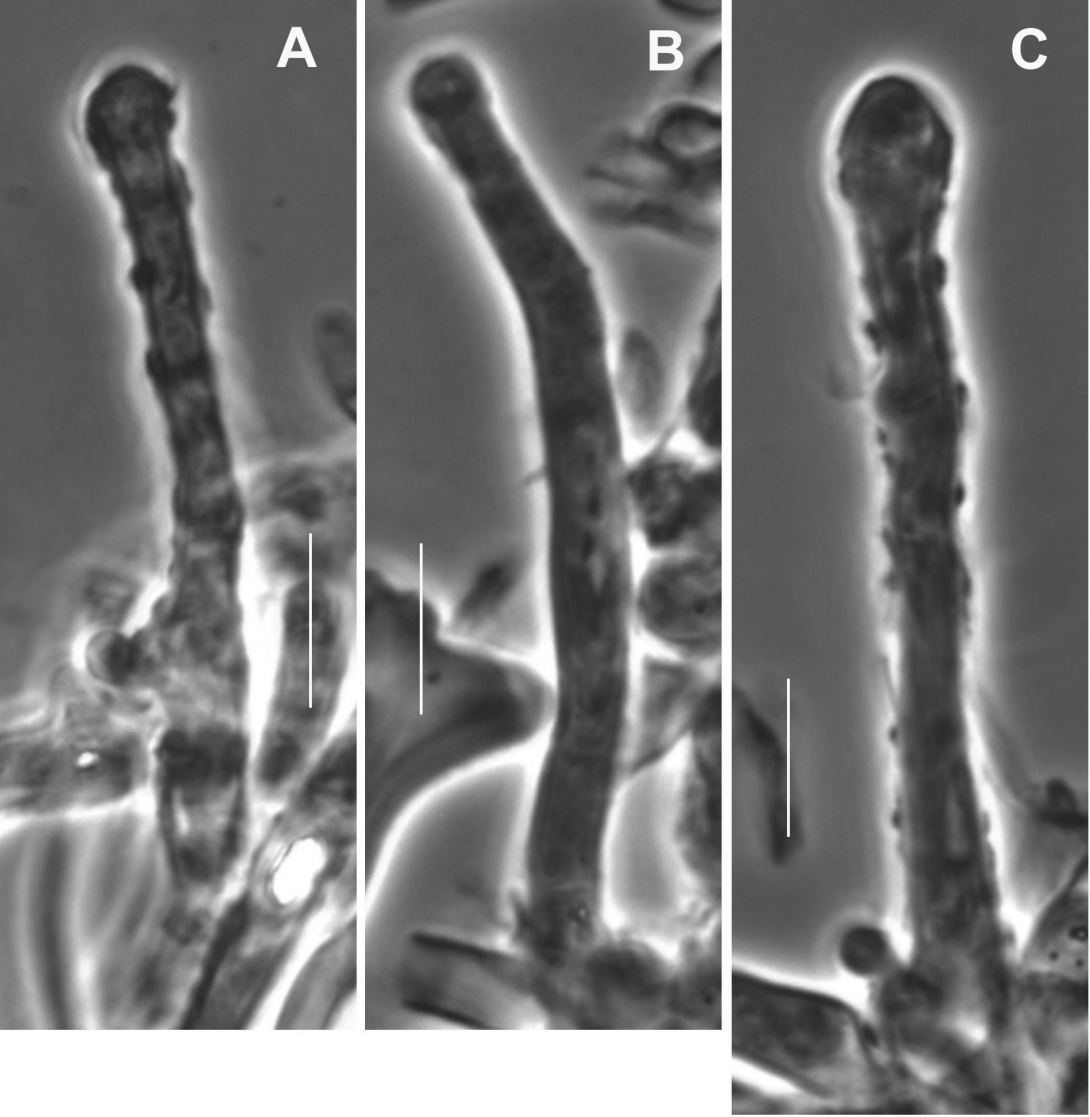
*Gymnopusportoricensis*. Pileal hairs. Note incrustation on thin slime sheath. **A** TFB 4512 (TENN-F-050999) **B, C** TFB 4548 (TENN-F-051029). Scale bars:10 μm.

**Figure 13.**
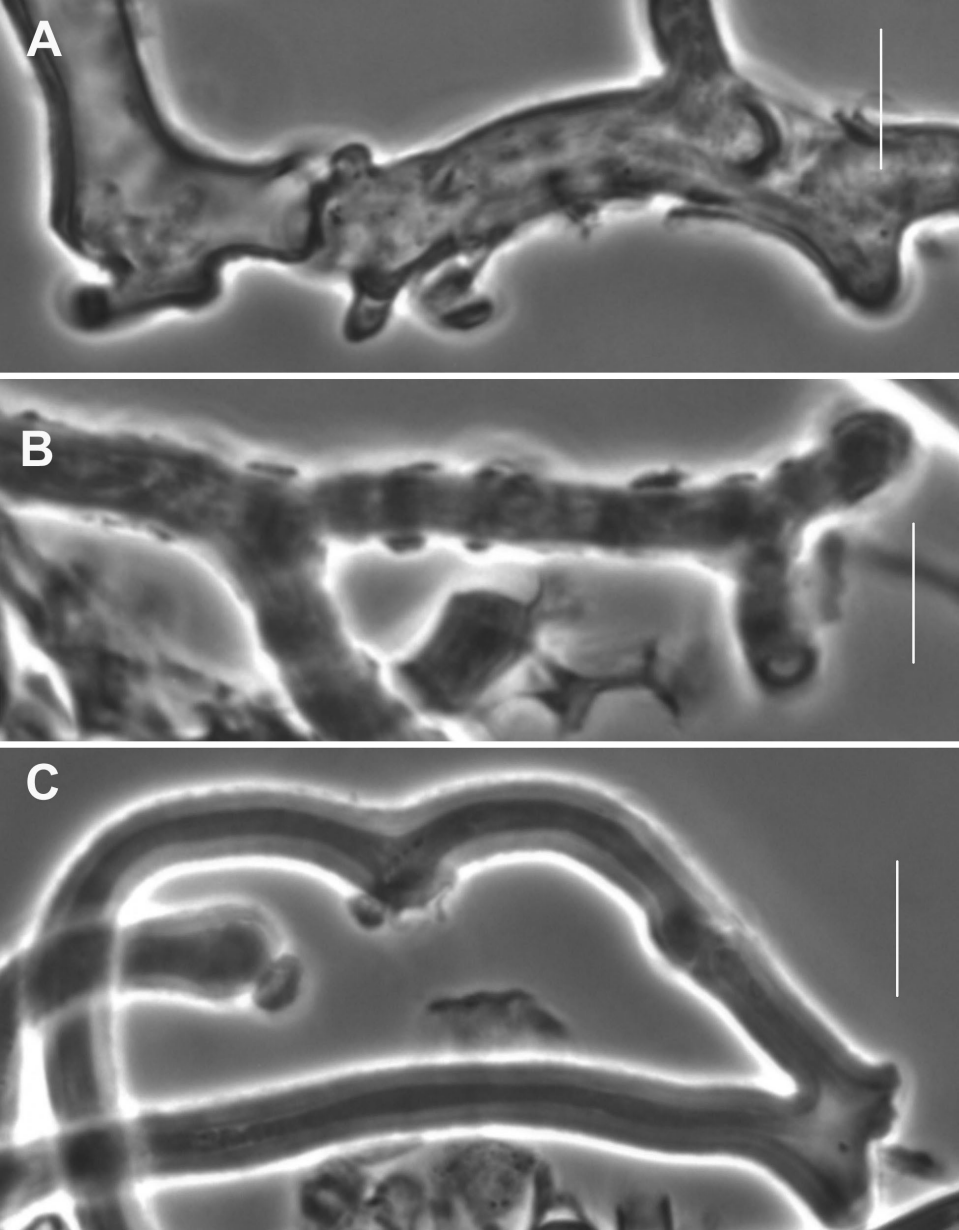
*Gymnopusportoricensis*. Pileipellis structures. **A** “Diverticulate” hyphal fragment **B** Encrusted hypha with thin slime sheath **C** Gelatinised hyphal walls. Scale bars: 10 μm. TFB 4548 (TENN-F-051029).

**Figure 14.**
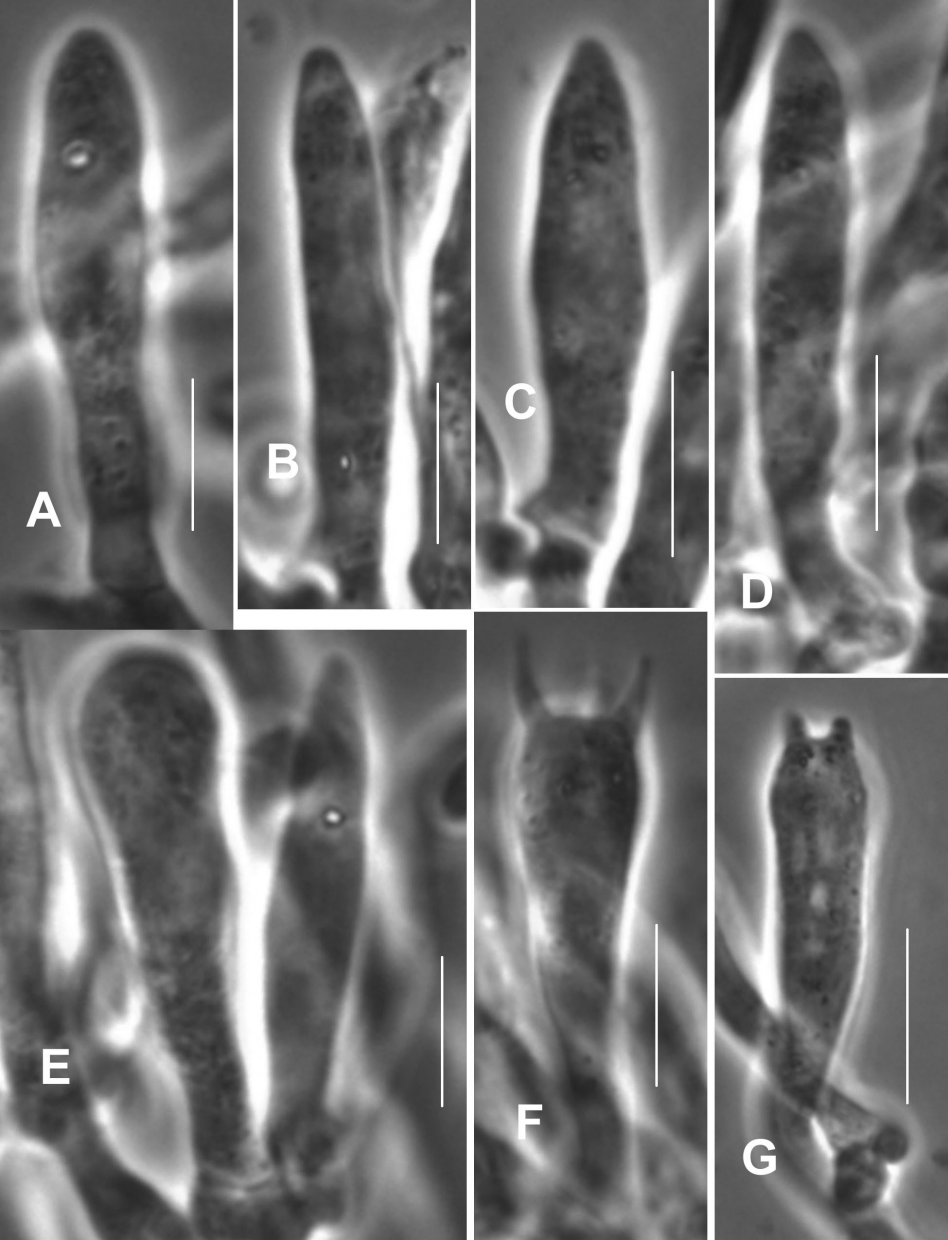
*Gymnopusportoricensis*. Hymenial structures. **A–D** Pleurocystidia **E** Basidiole and pleurocystidium from one clamp connection complex **F, G** Basidia. Scale bars:10 μm. TFB 4512 (TENN-F-050999).

**Figure 15.**
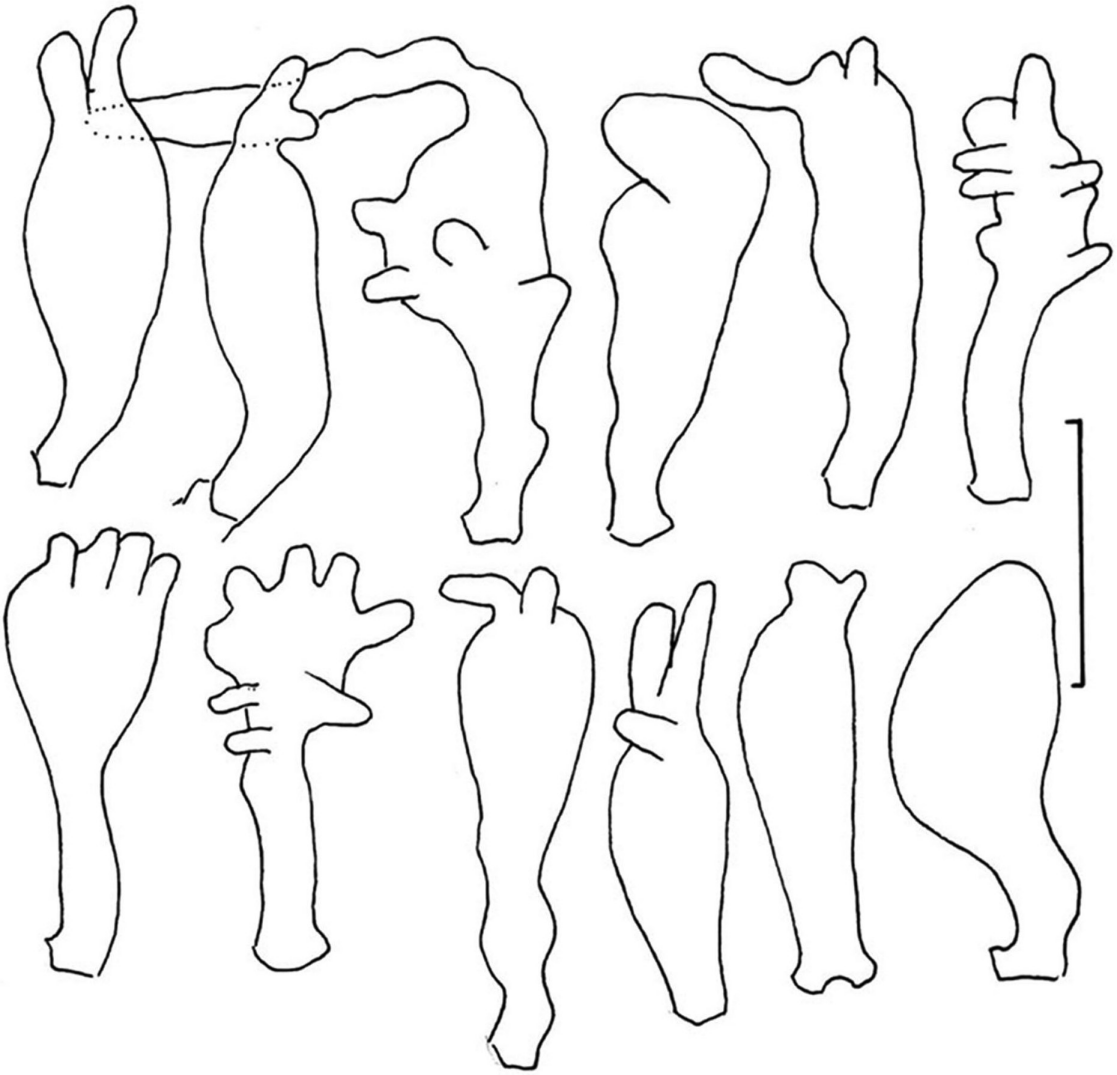
*Gymnopusportoricensis*. Cheilocystidia. Scale bar: 20 µm. TFB 4548 (TENN-F-051029).

Figs 10 , 11 , 12 , 13 , 14 , 15

##### Holotype.

United States, Puerto Rico, Caribbean National Forest, El Yunque, vic. Sabana, trail 3, 1.VI.1992, coll RHP, TFB 4548 (TENN-F-051029). GenBank: KY026628-9.

##### Etymology.

Portoricensis referring to collections made in Puerto Rico.

##### Diagnosis.

1) Basidiomata small, resembling those of *Gymnopusneobrevipes*, arising from rhizomorphs or from woody substrate, often in clusters of significant numbers; 2) stipe slightly eccentric or central, strongly curved, dark brown (black only at base); 3) rhizomorphs luxuriant, brown (not black); 4) spores somewhat small for the clade, (5–)6–7 × (2.5–)3–4 µm.

##### Description.

**Basidiomata** (Fig. 10) marasmielloid, cespitose to imbricate, conchate when young becoming shallowly convex to applanate by maturity, stipitate. **Pileus** 2–11 mm broad, circular to broadly reniform, matt, radially rivulose outwards, thin, leathery, uniformly “light pinkish-cinnamon” (7A2) to “pinkish-cinnamon” (7B5). **Lamellae** well-defined (-0.6 mm broad and ventricose to reduced, pleated or fold-like, distant (total folds = 11–18; through folds = 7–10), concolorous to pileus or “tilleul buff” (7B2); edge entire. **Stipe** very small (1–2.5 × 0.5–0.7 mm), slender, central or eccentric, strongly curved to non-instititious attachment on substrate (wood or rhizomorph), “Mikado brown” (7C6) apically, downwards “warm sepia” (7F6), “bister” (5F8) to black; basal tuft insignificant, blond. **Rhizomorphs** extensive, slender, brown, near “tawny olive” (5C5) or “sayal brown” (6C5) to nearly black. **Odour** and **taste** negligible.

##### Habitat.

Outer surface of old bamboo (TENN-F-051029) or rotting twigs of deciduous trees (TENN-F-050999).

##### Pileipellis

(Figs 11A, B, 12, 13) composed of three elements involved in very thin mucoid matrix: 1) hair-like, probably erect hyphal apices (Figs 11B, 12), 30–120 × 1.5–3 µm (at widest point), subtly capitulate apically, arising as side branches of slender hyphae (not from clamps), firm- but indistinct-walled, delicately decorated with gritty deposits or a very thin mucoid sheath, tapering to 1–1.5 µm diam. and subrefringent especially at very apex; 2) repent, heavily ornamented hyphae (Figs 11A, 13B) 3–9 µm diam., firm-walled, strongly encrusted in stripes or patches with no profile calluses; contents more or less homogeneous; 3) scattered rudimentary diverticulate hyphal apices (Figs 11A, 13A) 4–7.5 μm diam., often appearing stout-tibiiform, with diverticula lobate, 2–5 × 1.5–2.5 µm; contents more or less homogeneous. Pileus trama loosely interwoven; hyphae (Fig. 13C) 3–7.5 μm diam., conspicuously clamped, appearing thick-walled but gelatinised (wall -1.5 μm thick). **Pleurocystidia** (Figs 11C, 14A–D) 21–29 × 4–5 μm, fusiform, conspicuously clamped; contents homogeneous, occasionally subtly partitioned. Basidioles clavate, clamped; **basidia** (Figs 11C, 14F, G) 20–30 × 6–8 µm, 4-sterigmate, clavate, clamped; contents with scattered, minute guttules. Effete basidia do not disappear; at least the lateral walls survive to create debris in which turgid basidia are embedded in hymenial debris. **Basidiospores** (Fig. 11D) (5–)6–7 × (2.5–)3–4 µm (Q = 1.50–2.83; Q^m^ = 2.08; L^m^ = 6.58 µm), narrowly pip-shaped to sublacrymiform (somewhat tapered towards apiculus), thin-walled, smooth, inamyloid; contents homogeneous. **Cheilocystidia** (Fig. 15) limited to well-defined lamellae, scattered, 25–35 × 7–15 µm, pedicellate, thin-walled (easily crushed), expanded distally usually with irregular lobes or apical outgrowths, obscurely clamped, hyaline; contents more or less homogeneous. Stipe medullary hyphae of three types: 1) 6.5–24 μm diam., thick-walled, irregularly gelatinising [wall -1.2 μm thick in H_2_O, wall up to 7 μm thick in KOH and then yellowish (PhC)]; 2) 5–7.5 μm diam., thick-walled (wall -1 μm thick, not gelatinising, hyaline); clamp connections occasional, obscure; and 3) 2–4 μm diam., firm-walled, meandering through medulla; clamp connections rare, conspicuous. Stipe cortical hyphae 4–8 μm diam., strictly parallel, apparently adherent (held together adhesively and shattering under pressure), thick-walled [wall -2 μm thick, pigmented (ochraceous tan in KOH, red-brown in IKI/BF)], coarsely roughened in pigmented spicules; clamp connections not observed.

##### Commentary.

Although basidiomata superficially resemble those of *G.neobrevipes*, the pileipellis structure is not similar. Erect, broom cell-like cells of *G.neobrevipes* are missing; diverticulate repent hyphae are rare and doubtful; erect “hairs,” while clamped (and therefore assumed to belong to this organism), are more demonstrable in *G.neobrevipes*. Morphologically, *G.portoricensis* could be placed in *Marasmiellus* (see Retnowati 2018) based on poorly developed Ramealis-structure, no broom cells), but it equally could be interpreted as a reduced member of *Androsacei* (including *G.neobrevipes*) in which erect, broom cell-like pileipellis cells are rare to missing. Cheilocystidia are typical of the latter group. If *G.neobrevipes* is accommodated in Gymnopussect.Androsacei, *G.portoricensis* must also be found there. ITS sequences confirm this placement (Fig. 2).

Inspection shows that almost no basidiomata originate from rhizomorphs, instead seemingly originating from woody substrate directly. Rare basidiomata, however, do arise from rhizomorphs, with stipes as side branches. Moreover, some twigs with basidiomata are devoid of rhizomorphs altogether.

A polyspore dikaryon culture was established from TENN-F-050999 and careful examination revealed exceedingly rare (but clearly demonstrated) clamp connections. This condition is also true in cultures of *G.neobrevipes*. Desjardin (1990), while reporting clamp connections in the culture of *M.brevipes*, made no comment on their relative abundance.

Basidiomata are not pseudo- or eccentrically stipitate, but centrally to slightly eccentrically stipitate. The stipe, however, is usually immediately curved through the declivity in the pileus circumference. Lamellae appear to deteriorate rapidly, perhaps through insect grazing or tissue gelatinisation, but when discrete are shallow but sharply defined (not merely as folds). Interlamellar anastomoses are absent and even lamellar buttressing is missing. Instead, the interlamellar hymenophore is smooth.

These two collections fruited on very different substrata. The origin within bamboo structures would be difficult to imagine, so perhaps basidiomata arise from a very thin, arachnoid mycelium on the bamboo surface. Rare basidiomata were seen attached to rhizomorphs, which might support typical attachment to somatic hyphae.

If *G.portoricensis* is regarded as in *Marasmius*, the epithet (*portoricensis*) is preoccupied by *Marasmiusportoricensis* Murrill in Pennington. 1915. North American Flora 9(4): 262. The homonym is in *Marasmius* but not in *Gymnopus*. Described as having the longest ("longissimus") stipe – 6–8 cm × 0.5 mm – and pileus 4–10 mm broad, the holotype of *Marasmiusportoricensis* is at NY (isotype MICH) and the Mycoportal record shows several long-stiped basidiomata with stipe yellow-orange and apparently several long, straight rhizomorphs of similar colour.

An ITS-based clade (Fig. 2), which includes *Gymnopusneobrevipes*, *G.portoricensis*, two environmental sequences from Okinawa and a sequence of *Gymnopuscremeostipitatus* from Korea, is sister to the rest of Gymnopussect.Androsacesi. This section continues to expand with additional taxa yet to be determined and described.

##### Auxiliary specimen examined.

United States, Puerto Rico, Caribbean National Forest, El Junque, road to Verada Bisley, 18°15'53"N, 65°45'13"W V.1992, coll RHP, TFB 4512 (TENN-F-050999).

## Discussion

Singer (1948) proposed Micromphalesect.Rhizomorphigena based, in part, on his perception of gelatinisation of tissues in the pileus of the type species, *Marasmiuswestii* Murrill, (1945). Desjardin (1989) and Desjardin and Petersen (1989) concluded that diagnostic characters of *M.brevipes* matched those of *M.westii* and nomenclaturally, the epithet *brevipes* took priority. Moreover, these same characters more closely resembled those of Marasmiussect.AndrosaceiKühner (1933) than those of *Micromphale* and they transferred Singer’s section as Marasmiussect.Rhizomorphigena.

César et al. (2018) considered *Marasmiusbrevipes* and *M.westii* as taxonomic synonyms and transferred the latter as *Gymnopuswestii*. Based on our current examination of the type specimen of *M.westii* (FLAS-F-17211), we reject this synonymy. Some differences: 1) Hymenial elements are without clamp connections in *M.westii* while clamp connections are common in all tissues in *M.brevipes* (Desjardin and Petersen 1989 and this study); 2) pleurocystidia are not mammilate; 3) rhizomorphs are considerably thinner than those of *G.neobrevipes*; and 4) Murrill’s notes with the type of *M.westii* describe rhizomorphs as “aerial” (i.e. suspended above ground level) while those of *G.neobrevipes* are at or near ground level, predominantly bound to fallen substrate with some aerial elements.

## Supplementary Material

XML Treatment for Gymnopus neobrevipes

XML Treatment for Gymnopus portoricensis
